# Research impact of systems-level long-term care research: a multiple case study

**DOI:** 10.1186/s12961-017-0185-9

**Published:** 2017-03-21

**Authors:** Anita Kothari, Nedra Peter, Melissa Donskov, Tracy Luciani

**Affiliations:** 1 School of Health Studies, The University of Western Ontario, Labatt Health Sciences Building, Room 222, London, ON N6A 5B9 Canada; 2 Health and Rehabilitation Sciences, Faculty of Health Sciences, Western University, London, ON N6G 1H1 Canada; 3 Long-Term Care, Bruyère Continuing Care, Ottawa, ON K1N 5C8 Canada

**Keywords:** Research impact, Systems research, Long-term care, Case study, Integrated knowledge translation, Knowledge broker

## Abstract

**Background:**

Traditional reporting of research outcomes and impacts, which tends to focus on research product publications and grant success, does not capture the value, some contributions, or the complexity of research projects. The purpose of this study was to understand the contributions of five systems-level research projects as they were unfolding at the Bruyère Centre for Learning, Research and Innovation (CLRI) in long-term care (LTC) in Ottawa, Ontario, Canada. The research questions were, (1) How are partnerships with research end-users (policymakers, administrators and other public/private organisations) characterised? (2) How have interactions with the CLRI Management Committee and Steering Committee influenced the development of research products? (3) In what way have other activities, processes, unlinked actors or organisations been influenced by the research project activities?

**Methods:**

The study was guided by Kok and Schuit’s concept of research impacts, using a multiple case study design. Data were collected through focus groups and interviews with research teams, a management and a steering committee, research user partners, and unlinked actors. Documents were collected and analysed for contextual background.

**Results:**

Cross-case analysis revealed four major themes: (1) Benefits and Perceived Tensions: Working with Partners; (2) Speaking with the LTC Community: Interactions with the CLRI Steering Committee; (3) The Knowledge Broker: Interactions with the Management Committee; and (4) All Forms of Research Contributions.

**Conclusions:**

Most contributions were focused on interactions with networks and stimulating important conversations in the province about LTC issues. These contributions were well-supported by the Steering and Management Committees’ research-to-action platform, which can be seen as a type of knowledge brokering model. It was also clear that researcher-user partnerships were beneficial and important.

## Background

Long-term care (LTC) homes in Ontario, Canada, provide care for individuals requiring 24-hour assistance with the activities of daily living. The Ontario LTC sector includes 629 LTC homes which care for approximately 78,000 residents [1]. Resident complexity and demand is increasing due to the growing number of ageing seniors with multiple chronic health conditions. A systems-level approach is required to minimise systemic barriers to appropriate care planning and delivery, identify innovative solutions for the organisation of care, and provide sustainable improvements across the sector. In 2011, The Ontario Ministry of Health and Long-term Care (MOHLTC) established three provincial Centres for Learning, Research and Innovation (CLRI) in Long-Term Care to contribute toward enhancing the quality of care in the LTC sector through education, research, innovation, evidence-based service delivery and knowledge transfer. This study focuses on the systems-level research projects associated with the Bruyère CLRI in LTC. We were interested in understanding the research contributions arising from these systems-level research projects during the research process, in advance of anticipated outcomes, materials and knowledge products. Therefore, this study focuses on five systems-level research projects which were either still at the research formulation phase or early in the data collection stage.

The impact of research processes and contributions is of increasing interest, especially in the current environment where funders and governments are demanding increased accountability by way of research products that lead to health outcomes [2]. The literature in this area has received much attention of late, resulting in the development of research impact frameworks like the popular payback model, research impact model, research utilisation ladder, Weiss logic model, health technology assessment organisation assessment framework, societal impact framework, balanced scorecard, and cost-benefit analysis [3]. Individual studies about the benefits of research are diverse in scope; for example, some have focused on the impact of certain datasets or disciplines [4, 5], countries [6, 7] or grants [8, 9]. A pivotal moment for the field was the 2014 Research Excellence Framework in the United Kingdom, where higher education institutions were asked to provide data related to impact indicators that were in turn tied to resource allocation and benchmarking [10]. This experience, along with a number of review articles [11–18], raises some key issues related to conducting impact evaluations, including that of bias (are the assessors also the funders of the impact evaluation, or are they the researchers themselves? is there recall bias related to retrospective reporting?), the high cost and labour intensiveness of evaluations, the minimal attention paid to uncovering policy impacts, and the heterogeneity in defining and measuring research impact. The translation of research findings into policy and practice outcomes is a complex, dynamic process with extraneous factors that make it difficult to claim casual links between research and its use [19].

Inviting research user input in the early stages of a research project can better ensure that the work is mutually beneficial, leading to increased use of research findings [20]. In particular, creating space for multiple perspectives to guide a project is necessary to identify appropriate and feasible solutions for complex LTC problems, like those addressed by the five systems-level research projects. Kothari and Wathen [21] discuss the increased involvement of practitioners, administrators and policymakers in research through integrated knowledge translation, where partners collaborate throughout a research process to shape research questions, interpret data and disseminate research findings. This bridging of researcher and practitioner/policymaker worlds is expected to lead to research questions characterised as having real-world relevance with heightened potential for research uptake. Other valuable outcomes related to the enhanced relationship between the involved parties include a better understanding of each other’s roles and a shared worldview about the problem at hand [21]. A number of promising approaches, such as contribution mapping, the Spirit Action Framework and the participation research impact model, take up the idea of user engagement in the context of research impact assessment [2, 22].

The Bruyère CLRI takes a unique, hands-on approach to supporting systems-level projects through their CLRI Management and CLRI Steering Committees, which help to guide the research. Specifically, the CLRI Management Committee consists of five senior CLRI members who meet monthly to discuss ongoing and potential research projects. The Committee is charged with ensuring projects are progressing (based on work plans that include milestones and deliverables), making decisions about resource allocation and facilitating links to relevant stakeholders. The Committee also plans events and discusses dissemination activities, such as identifying conferences at which project findings can be shared. The CLRI Steering Committee is comprised of 11 key stakeholders from the broader LTC sector (e.g. health planners, associations, educational institutions, family/resident councils, LTC homes, etc.). The Steering Committee ensures that the knowledge and tools developed through the CLRI are relevant, practical and valuable to the LTC sector.

Research benefits arise at the individual, organisational and system levels. Research can also have effects throughout the research process. For example, the research capacity of those involved in a research project can be developed as the research takes place. Similarly, knowledge about a substantive content area is gained through participation in the research process. These outcomes may be particularly relevant when the research project is designed as a partnership between researchers and research end-users (e.g. policymakers, practitioners, administrators).

The study research questions were:

1. How are partnerships with research end-users (policymakers, administrators and other public/private organisations) characterised?
2. How have interactions with the CLRI Management Committee and Steering Committee influenced the development of research products?
3. In what way have other activities, processes, unlinked actors or organisations been influenced by the research project activities?

## Methods

### Study design

This research used a multiple case study methodology, which supports the investigation of socially complex phenomena and its surrounding context, thereby providing a deep, holistic understanding of phenomena [23]. Each of the five LTC systems projects was treated as an individual case; they are described in Table 1.

**Table 1:** Description of systems level research projects

	Background	Objectives	Methods	Timelines	Anticipated outcomes
1. Health Human Resources Forecasting Model	- Approximately three-quarters of direct care staff in Ontario’s long-term care (LTC) sector are Personal Support Workers (PSWs) - Despite their importance, relatively little is known about them	To create a dynamic forecasting model to outline the current and future use of PSWs and nurses in the LTC sector	- Through three phases, build and validate a forecasting model that brings together demand and supply data for PSWs and nurses	Phase 1: 2014 Phase 2: 2015 Phase 3: 2015–2017 (Ongoing)	- Knowing how many nurses and PSWs will be required, as well as the projected supply, will assist provincial planning
2. Improving Wait Times and Transitions in Care	- Concern about the huge (alternate level care) numbers of patients in hospitals - Backlogs exist while patients wait in one facility for a bed to become available in another facility	To provide a queuing model that maps how patients move through a network of post-acute services (including LTC) in order to better understand the necessary capacity at each node in the network	- Development of a network queuing model with blocking, development of a simulation model, comparison of the simulation results with current performance metrics, optimisation and testing of the model	2013–2015	- Provide insight as to the average build-up of clients waiting at each facility and what alternative facility they are seeking to enter - Determine optimal capacity and resource allocation for the various facilities that minimises blocking at each stage
3. Understanding Health Care Use and Cost for LTC and End-of-Life	- Better understanding of the major population drivers of LTC admissions and outcomes, as well as the cost drivers for LTC services required	To examine factors that influence healthcare use and cost, and reporting on indicators that measure the performance of the healthcare system at the end‐of‐life and in LTC	- A retrospective, 12-month analysis of several variables for all deaths in Ontario in 2011 and 2012 (177,817 deaths)	2013–2015	- Understand the role of socio-demographic variables, co-morbidity, health system variables and acute healthcare events on healthcare use and costs at the end-of-life and in LTC
4. Specialised Units in LTC Homes	- Despite the potential of Specialised Units and the well-documented gaps in the current care system, the number of designated Specialised Units remains low	To create a toolkit for LTC Homes interested in establishing a Specialised Unit; explore capacity planning aspects of LTC Specialised Units in Ontario; and assist interested LTC Homes with their application for a Specialised Unit	- Review existing literature and other information on providing specialised care to LTC residents - Province-wide consultation with stakeholders about their experience with Specialised Units	2014–2017 (Data analysis ongoing)	- Better understanding of the facilitators and challenges for Specialised Units - Multi-stakeholder toolkit to support the designation process
5. Framework for Case Costing in Long-Term Care	- Case costing is critical to plan for efficient models of care delivery and for decision-making; although it is common in other healthcare sectors, it is not practiced in LTC	To develop a case costing framework for the LTC sector and to generate an approximation of resident-specific costs in two LTC homes	- As a first step, review the literature to identify (1) case costing frameworks in LTC and/or other sectors, and (2) nursing workload proxies for LTC; consult with experts in the field	2013–2015	- Enhanced decision-making for resource allocation and staffing based on the actual resource requirements by residents, not purely based on historical staffing models

A recent framework by Kok and Schuit [24] focuses specifically on research impacts generated through integrated knowledge translation processes by researchers and research end-users throughout the research process. Entitled contribution mapping, this model for research monitoring and assessment focuses on the actors who are involved in, or directly interact with, a research project and assesses contributions of research rather than difficult-to-ascertain impacts. Contribution mapping may be useful as it promotes learning and reflection by the ‘linked actors’ and stimulates further efforts to enhance the contribution of research [25]. This framework distinguishes among four categories of research-related contributions. Category one refers to changes in the ability and actions of the investigators and those associated with the research (‘linked actors’) that occurs in research project activities. Category two refers to the knowledge products that are created and presented or stored in a systematic way; these may include scientific and other publications, new research projects, protocols, methods or equipment. Category three refers to the contribution of knowledge-to-action processes such as becoming a part of routine practice, a component in successful innovation, or an element in decisions and decision implementation. Category four refers to the contribution of knowledge-to-action by actors who are not involved or linked to the research project (‘unlinked actors’); this fourth category is described as difficult to identify and verify as it is much more problematic to access. What is novel about this framework is its focus on changes in ability (skill development or capacity) and its attention to actors outside of the research partnership. Therefore, the contribution mapping framework guided this work.

### Sample

Research end-users (e.g. government and other contacts) were identified by each project team and in consultation with the knowledge broker at the Bruyère CLRI. The knowledge broker also identified other potential participants at Bruyère, including members of the research teams, Steering and Management Committee members, and individuals not linked to the projects. This purposive sampling strategy allowed a diversity of opinions to emerge. Potential participants were invited to take part in the study via an email sent from the research team; an information letter was also sent as part of the written informed consent process.

Adult participants who spoke English and were connected to one of the systems-level research projects being studied at the Bruyère CLRI were eligible for participation in this study. In this case, ‘connected’ meant one of two things, namely (1) a member of the research team for the projects, or (2) an end-user of the research project. Other key individuals and unlinked actors were also of interest.

### Data collection

Two rounds of data collection, in April 2015 and February 2016, allowed for in-depth examination of the research project, as is characteristic of case study approaches. It also accommodated the development of research partnerships and related activities during the intervening time period. Since this case study was concerned with examining research contributions and impact during the research process, no further data were﻿﻿ to be collected following this time period.

Research teams, the Steering Committee and the Management Committee attended their respective focus groups at the Bruyère CLRI offices or remotely through a secure telephone conference line. In most cases, the focus groups were smaller than the recommended six to eight participants, but it was felt that homogenous groups that stimulated rich, relevant discussions were more important than combining different groups (i.e. different research teams) for a larger-sized focus group. Research users and unlinked actors were interviewed by phone. The first and second author conducted the focus groups and interviews; the third and fourth authors had overlapping roles as members of either a research team or one of the Committees, and thus served as focus group participants in some cases. The moderator’s guide for the research teams (Box 1) asked about the structures and processes related to the research team-user partnership, the interactions related to the research team and the Committees, perceptions of quality related to these relationships, challenges, and outcomes. These questions were adapted for the other respondents as necessary. Greater emphasis and time were devoted to discussing outcomes during the second round of data collection.

Box 1 Focus group moderator guide for research teams

**Table:** 

1. Please tell me about project X and your respective roles in the project. Who else is on the team? What stage is the project at now?
2. How are roles and responsibilities of the research team, the CLRI Steering and Management Committees, and the research end-users established?
3. As a research team, how did you build collaborations/partnerships with research end-users? What is your history with them?
4. What strategies have you used to ensure that the research being conducted is relevant to the research end-users?
5. Can you tell me about the collaboration between yourselves, other CLRI researchers/teams and the CLRI committees over the course of the project thus far?
6. What communication processes do you have in place between the research team and the research end-users? The CLRI committees? How effective do you find these processes?
7. What structures do you have in place for research end-users and the committees to provide meaningful insights and guidance for the research project?
8. Do you feel that research end-users and/or CLRI committees have been able to do this so far in the research process? Why? Can you give an example of how their input has impacted your project?
9. Let’s talk about the quality of the relationships within your collaboration. Could you talk about what a quality relationship means to you? Based on that definition, tell me about the quality of relationships you think you have with members of CLRI committees/research end-users?
10. Are there ways in which you think that the quality of relationships could be improved? Are there any challenges in the research partnership regarding whether and how you use research and knowledge and how you collaborate with one another? If so, please describe how this usually happens, its impacts and whether/how disagreement is resolved.
11. Do you think your work with project X, in collaboration with the CLRI committees and research end-users, has impacted you as a professional? Why or why not? If so, how?
12. Have you shared learnings/information from the research project with others in your academic institution or organisation? Can you tell me about that?
13. Are there people or organisations outside the direct sphere of the research project (e.g. not part of the research team, end-users or committees) that you feel have been impacted by this research project?
14. What do you feel Project X can contribute to the long-term care sector?

Research participants and the Bruyère CLRI Management Committee were asked to identify relevant documents that described project activities. These documents consisted of meeting notes, ethics board applications, project summaries and presentations. Documents were used to provide contextual information about the cases. The first round of data collection involved interviews and focus groups from research team members (n = 12), key individuals at Bruyère and the CLRI Management Committee (n = 4), end-users (n = 4), and the collection of 37 documents. During the second round, data were collected from research team members (n = 7), the CLRI Management Committee (n = 4), CLRI Steering Committee (n = 5), end-users (n = 6), other unlinked stakeholders (n = 1), and from 27 documents. A total of 261 pages of participant transcripts were analysed.

Preliminary findings from the first round of data collection were presented at a provincial conference hosted by the Bruyère CLRI, serving as a type of face validity of initial results by LTC sector representatives (including some research participants). The same findings were also presented to the Steering Committee before starting the second round of data collection. In both cases, the audiences expressed an interest in the work and found the results interesting, indicating that the preliminary findings were credible.

### Analysis

Focus groups and interviews were recorded (to support accuracy), transcribed professionally and de-identified before being imported into the qualitative data management software NVivo 10. Documents were also imported into the software. Each case – each of the five research projects – was first analysed independently using related transcripts (from the research teams, their specific research-user partners and unlinked users) and associated documents. The ways in which these cases were influenced by, and in turn influenced the Steering and Management Committees, was also of interest.

The analysis of texts was informed by framework analysis to support the development of inductive and deductive themes arising from participant data as well as the research questions [25–27]. The second author read all the transcripts and coded a few transcripts to develop preliminary codes. The first and second author came together to review the data and preliminary codes in order to develop an initial thematic framework. Through iterative coding and discussion, themes and sub-themes evolved using examples from the data.

Each case was analysed holistically to understand the nuanced contributions developing from each research project, and tables that summarised the data related to each case were used to conduct the cross-case analysis. All research team members met to interpret themes through the exploration of alternative explanations; team members’ diverse disciplinary perspectives ensured the data were being interpreted reasonably. Consensus on interpretation was achieved through discussion.

## Results

There were many benefits to working with end-users in an integrated knowledge translation fashion, as well tensions between balancing scientific needs with research user needs. Meanwhile, the Steering Committee had an important role in bringing the views of families, LTC residents and other sector stakeholders to the research teams. The Committee’s ability to understand both policy and research needs acted as a bridge between these two worlds by connecting researchers to appropriate stakeholders and other resources. Through all these processes, along with the merits of the research being conducted, there was evidence that the projects made contributions through advancing knowledge, contributing to changes in understandings about topic areas, building capacity and developing user-friendly knowledge products like toolkits. Of particular note is that the projects were on-going, demonstrating that project outcomes and impacts can occur even before the completion of research work. The thematic findings related to each case across both data collection periods are presented in Table 2.

**Table 2:** Findings by case

Case	Dimension	Themes
Health Human Resources Forecasting Model	Centre for Learning, Research and Innovation (CLRI) Involvement	Defining the research questions and scope of the project
		Opening doors and introducing relevant stakeholders
		Providing the opportunity for involvement/collaboration in additional projects
	Partnership Development	High level of interest from the Ministry
		Grant opportunities and research collaborations
		On-going consultation with relevant stakeholders to integrate their needs and feedback
	Influence on Other Activities	Trainee development
		Work was leveraged for additional resources and funding
		Opened up conversations with the Personal Support Worker sector
Wait Times and Transitions in Care	CLRI Involvement	Funding of the project was important
	Partnership Development	Stakeholder consultation helped to tailor the product
		Helped researcher articulate challenges with the modelling
		Easy to work with front-line partners, more challenging to work with management
		Researcher credibility/methodological credibility difficult to establish because of model complexity
		Working with partners provided a better understanding of the user and the user context
	Influence on Other Activities	Project lead to Natural Sciences and Engineering Research Council of Canada grant
		Presentations that would not have otherwise happened
		Initial conversations (interest) from the Ministry
Healthcare Use & Costs at End of Life	CLRI Involvement	Recognised researchers as content experts
	Partnership Development	Will do sub-analysis or focused analysis for partners
		Working with partners provided a better understanding of the user and the user context
		Share manuscript drafts for validation of findings
		Finding balance between user needs and academic knowledge needs
		Discussions with partners to determine if new indicators are acceptable
		Challenging to interact with Ministry due to its size
	Influence on Other Activities	Media attention (due to controversial results)
		Stakeholder engagement (due to controversial results)
		Article in *JAMDA*, plus a number of other scholarly publications (about 8)
		Phase 1 data collection invoked reflection on policy-oriented project research-users and their needs
		Indicators for end-of-life care incorporated into quality improvement plans and other stakeholder reports
		Collaborate with researchers who want experience working with long-term care (LTC) and home care data (capacity building)
		Collaborate with another province
		Trainees
		Led to Canadian Institutes of Health Research grants; also collaborative grants with other CLRIs
		Online web tool
		Although project is aimed at system level, get calls from organisational/facility level
		Shifting the conversation at Health Quality Ontario from facilities to system-level measures
Specialised Units	CLRI Involvement	Engagement with diverse perspectives through the Steering Committee
		Linkages and connections made through Management Committee
	Partnership Development	Stakeholder consultation changed original direction of product
		Stakeholder consultation helped to tailor the product
		Working with partners provided a better understanding of the user and the user context
		Raising the profile of designated units at the Ministry and provincially
	Influence on Other Activities	Raising awareness about what a designated unit is (general education)
		Raising awareness about units as provincial resources
		Making connections between stakeholders, as requested (at conferences, webinars)
		Trainees
		Toolkit developed
Case Costing Framework	CLRI Involvement	
	Partnership Development	Exploring feasibility of project is taking time
		Discomfort from some around identifying costs in LTC
	Influence on Other Activities	Leveraged the extra fellowship
		Sparked conversation in the sector
		Asked to present at some conferences

### Description of cases (also see Table 1)

Here, we first provide a brief summary of the cases, followed by a presentation of four higher order themes from the cross-case analysis.

1. Health Human Resources Forecasting Model – This study aimed to create a forecasting model which outlines the current and future use of Personal Support Workers (PSWs) and nurses in the LTC sector. Approximately three-quarters of direct care staff in Ontario’s LTC sector are PSWs. Through three phases, this project builds and validates a forecasting model that brings together demand and supply data for PSWs and nurses. On completion of this current study, this project team had developed a model, presented preliminary forecasts from the model, and was working on extending the model to other health sectors.
2. Improving Wait Times and Transitions in Care – Based on the concern about the huge alternate level care numbers of patients in hospitals, this project focused on creating and optimising a queuing model that maps how patients move through a network of post-acute services (including LTC) in order to better understand the necessary capacity at each node in the network. On completion of this current study, a simulation model had been developed and tested demonstrating that severe blocking occurs for patients seeking entry into LTC, while other nodes in the network are seen to flow more smoothly.
3. Understanding Health Care Use and Cost for Long-Term Care and End-of-Life in Ontario – This study was part of a series of projects that describe end-of-life care and LTC in Ontario, including an examination of factors that influence healthcare use and cost, and measuring the performance of the healthcare system at the end-of-life and in LTC. A retrospective, 12-month analysis of several variables for all deaths in Ontario in 2011 and 2012 (177,817 deaths) was undertaken. On completion of this current study, this project was completed, and a breakdown of costs, including percentages of cost allocated to each of inpatient care, primary care, home care, LTC and palliative care, was provided.
4. Specialised Units in LTC Homes – Following a provincial multi-stakeholder consultation, this project aimed to create a toolkit and provide facilitation to transfer Bruyère LTC expertise in creating specialised units for use by other stakeholders and LTC facilities. The methods for this study included a review of existing literature and other information on providing specialised care to LTC residents, followed by province-wide consultation with stakeholders about their experience with Specialised Units. On completion of this current study, this project was collecting data.
5. Framework for Case Costing in Long-Term Care – This project focused on the development of a case costing framework for the LTC sector and generating an approximation of resident-specific costs in two LTC homes. Case costing is critical to plan for efficient models of care delivery and for decision-making. The methods included reviewing the literature to identify case costing frameworks in other sectors, uncovering nursing workload proxies for LTC and consulting with experts in the field. On completion of this current study, this project had been concluded due to the absence of required data. It was concluded that, in order to implement case costing within LTC, it is essential that a method for workload measurement be developed.

### Cross-case results

#### Benefits and perceived tensions: working with partners

The data revealed that there were many benefits to working with research end-users in an integrated knowledge translation fashion. Early input ensured the right research questions were being asked such that they were geared to the system level. On-going discussions were equally useful in that assumptions and limitations associated with research methodologies (or datasets) could be made transparent. Research products were tailored to partner needs, as is characteristic of co-produced knowledge. In a few cases, the research product changed completely from its original conception based on the contributions of partners. For example, in the specialised units project, initially, a toolkit was to be developed for LTC homes but once the research got underway it became clear that perhaps the Ministry, who is responsible for determining which LTC homes are approved for specialised units, may benefit from a report on key findings. The direction of the case costing project was also changed due to the concern from partners about its feasibility.

The individual cases demonstrated that two major forces drove partnerships, namely the need for administrative data and in response to external motivations. In many cases the relationship between researchers and end-users was chiefly characterised by the researcher’s need to access administrative data. Thus, end-users played a critical role in providing this support. In exchange, end-users received research results that were relevant for their organisations.

Relationships also developed in response to external motivations. For example, researchers responded to organisational issues that were important to end-users, or researchers spoke about collaborating on topics that were important in the LTC sector at large or in anticipation of upcoming issues. Maintaining this applied focus was seen as an important way to garner interest and support, but also meant remaining flexible in order to respond to changing needs. Researchers were also motivated to formulate research topics that could be relevant to expected policy needs. As one research team member stated, the current status of LTC is unsustainable, which was interpreted as meaning that policy changes and sector reform were forthcoming and required. Both researchers and their partners wanted to contribute knowledge that would inform those upcoming policy changes. Grant opportunities and collaborative submissions were another common external motivation for partnership development.

Nevertheless, working with partners also presented tensions in some cases. One of the tensions that emerged repeatedly was the need to balance scientific needs – both in terms of knowledge gaps in the literature and academic promotion – with needs identified by research users for their organisation or for sector planning. In one case, this resulted in provocative results being published in an effort to be true to the data, with the consequence of much media coverage and visibility among the LTC community:

“*We had to balance the fact that obviously some people in the LTC sector might not want us to look at that angle, but we have to balance that against what’s academically relevant and most publishable right…*”

Another fall-out of this tension was that complex research approaches to solving the identified problem may not always be appreciated or understood by the user group. For example, solving these complex questions can require complex inter-disciplinary methods and concepts which can be difficult for partners to understand. Perhaps relatedly, there was some thought that some researchers were not taking full advantage of the linkages the CLRI provided. For example, sometimes a contact name was provided but minimal follow-up occurred.

The analysis revealed another minor tension. The process of giving feedback was seen as time consuming by some research users. They found it difficult to determine if their time was well spent, if the input was used correctly, and more generally “*what pieces were incorporated or not incorporated and for what reasons?*” Underlying this point was the understanding that significant changes may not be possible given the stage of the research and/or the larger context.

The funder, MOHLTC, was not available to participate in this study. A few study respondents mentioned their experiences in working with this partner. First, relationships were described as being ad hoc in that there were no regularly scheduled meetings. Meetings often focused on results and next steps once findings became available. One researcher acknowledged the need for a more formal interaction:

“*I think when they* [MOHLTC] *hear about our project, they have been very interested, but to have a regular table where we can actually meet and share directly from researchers to ministry staff, I think that would be something that we can improve in the next round* [of the CLRIs].”

Second, the Ministry was perceived as quite large, making it difficult to navigate and determine who would be most interested in the work. In one case, it was felt that the Ministry did not have the receptor capacity to deal with the project findings at that point in time due to competing priorities. Largely, however, relationships with the Ministry were not a prominent point of discussion in the focus groups, apart from mention of some presentations. This is important to highlight in the face of systems-level research needing to influence policymaking at the highest levels.

#### Speaking with the LTC community: interactions with the CLRI Steering Committee

The CLRI Steering Committee was very much seen as bringing the community back into the research conversation. The Steering Committee is made up of diverse stakeholders, some of whom acted as advocates for the families of residents, a perspective that had minimal articulation in previous systems-level research. The voices of the broader LTC community were also conveyed through the Committee to the research projects, in effect “*providing some kind of external accountability process to the work of the CLRI*”. The Committee brought a valuable humanistic perspective to the complex (and mostly quantitative) projects. In addition, the Committee brought information about the context of LTC homes or the general LTC system to overcome the sense that “*there was a lack of connection with the real world*”.

Feedback from the Committee mostly happened at the early stages of a research project, but structures were in place at regular meetings for project updates and input. The Committee felt that their comments were being heard; they could see a shift in the research or in presentations about the research. On account of this interaction, researchers were able to better anticipate and address issues that might come up from other stakeholders. Researchers were also better able to represent the generalisability of the work, “*defining more clearly where it will be useful and where it might not*”.

Through this back and forth there was also a sense that the Committee provided support for the research, and became sensitised to issues in the LTC sector, bringing this worldview to their conversations with other important stakeholders, like Ministry tables. The Committee disseminated the project findings through their own networks and members, extending the reach of the research. The knowledge that Committee members gained was often brought into their own professional activities, like curriculum development. There was a feeling of teamwork and community in that a LTC home was not alone in dealing with sector issues. Some Committee members felt they could be even more engaged with dissemination and implementation, or with further direct contact with the researchers.

#### The knowledge broker: interactions with the management committee

The roles and function of the Management Committee were characteristic of a knowledge broker, or someone who can speak the language of both research and policymaking/practice. In fact, most Committee members were also members of research teams. As a knowledge broker, the Committee ensured that research and policymaking needs were translated or communicated appropriately to the other side so that they could be addressed. Their “*responsibility is to do the actionable work that can change policy or result in implementation*.” The Committee also acts as a mediator to the public through the media. When necessary, they got involved following the publication of a research project’s findings:

“*I guess I kind of look at this as we’ve been commissioned by government to carry out a set of responsibilities and we in turn have then commissioned folks to do projects that we’ve approved, and so we’re the intermediates between the reporting of the findings that we found … if there’s a problem in either reception, interpretation, whatever, yeah I think it’s our responsibility to get in front of that*.”

In this capacity, the Committee tries to anticipate reactions, eases any tensions and helps communicate the results accordingly.

In the more traditional role, the Committee advocates for research projects in the LTC sector, but even that task needs to be done carefully given that the Committee has built multiple partnerships that need to be maintained. An important task is nurturing the partnership with the Ministry. Generally, however, the Committee raises the profile and the ‘visibility’ of the research through its multiple contacts within the sector. The Committee plays a direct role in dissemination of research through the organisation of webinars and conferences.

Internally, the Committee assists researchers by providing advice about methodology through: critical discussions; regular contact with researchers to determine their progress and needs (like datasets); and solutions to problems (e.g. obtaining a visa for a postdoctoral trainee or obtaining permission to use datasets). One of the most important things the Committee does for researchers is make connections with stakeholders within and beyond the LTC sector who might be interested in using the project findings. This connection might be in the form of direct project linkages, or it might be to initiate presentations to a specific or broader audience in the sector. Linkages are also made across jurisdictions, with other universities or the private sector. Often, requests for assistance come from outside Bruyère, in which case the Committee makes the introductions to the appropriate researchers. Multiple times researchers said that these connections were invaluable for their research, and for the utilisation of the work. Nevertheless, there is variability in terms of “*grabbing the bull by the horns and running with these linkages*” in that some research teams were better at optimising these relationships and capitalising on opportunities.

#### All forms of research contributions

One of the fundamental ways that the projects made contributions was through advancing knowledge, which at times included dispelling existing misconceptions about research topics. The benefits of productivity were being enjoyed as many projects had already generated scholarly publications, although not all were at this stage yet. Numerous presentations were also made to the LTC community. Respondents noted that it was important for research to have both academic acceptance as well LTC sector acceptance.

Specific changes in understanding about a topic were also documented. Such changes were not the result of particular dissemination efforts, but rather came about through participation in or learning about the research process. These are valuable for stimulating specific conversations in the sector that were until now not receiving much attention. One stakeholder was particularly enthusiastic,

“*…it’s been such a positive experience I, professionally, I would be less hesitant to reach out to researchers. In the past I don’t know that I would have responded to even participating in this interview, but I’ve had such a positive experience with Bruyère that I was more than willing to respond to it*.”

The iterative nature of the integrated knowledge translation process was acknowledged by at least one partner, who said “*well I just think by sharing the results as they’re going, I think that’s been very beneficial in that we don’t have to wait until the very end to see how it’s going.*” Some partners also benefited from these relationships by being able to obtain targeted evidence for their own decision-making. This was described as, “*…help*[ing] *them advance their cause, you know, by pulling data out, pull*[ing] *information out that might be helpful to what they’re trying to do*”. Regular meetings (a key characteristic of the integrated knowledge translation approach) also meant that projects were discussed regularly by partners within their own planning meetings: “*This particular research comes up probably monthly when I’m reviewing with senior directors and team leads and senior management within our* [organisation].”

At this early stage, one visible contribution was in the area of capacity building, which is important because optimal capacity sets the stage for future research. This includes such things as building research relationships across provinces or with researchers in other sectors, or teaching researchers about effective knowledge dissemination. Researchers were also appreciative of the interdisciplinary teams that were deliberately encouraged by the CLRI. As one researcher expressively describes:

“*…this was under the umbrella of the CLRI so for sure they have brought together people. I think that’s the biggest challenge in research right now. Everyone gets focused on their one question, their one job, and then they keep asking further questions…until sometimes you go down a rabbit hole and…it’s good. Collaborations, what they do is they take you back out of the rabbit hole and get you to look at things in the bigger more holistic perspective and I think that’s always healthy for researchers…*”

This approach built a different type of research capacity among the academics. Training of highly qualified personnel, and student development, was also characteristic of these projects.

Other tangible contributions were identified as user-friendly bilingual (English and French) toolkits or online resources, which were seen both as practical tools for specific users, like administrators, but also as further attempts to raise awareness about topics. Participants also mentioned that some project outputs, namely indicators, had been incorporated into other provincial level quality improvement plans, accountability agreements and reports. It was clear that some contributions, be it to policy or products, were under discussion with relevant stakeholders like the local planning body. As one participant noted, “…*we’re still in the early stages of trying to figure out how to best use the data. And how to use it to drive improvements*”. Project influence was also seen in the interest from unanticipated people or organisations not originally targeted as users (unlinked actors). Of particular surprise was the interest from individual LTC homes.

## Discussion

This study identified contributions from systems level research projects in advance of anticipated outcomes, materials and knowledge products. This project uncovered nuances related to research contributions that occurred at the early stages of research, which may not be uncovered in typical impact measures. Here, we raise three high level, interrelated points of discussion drawn from the cross-case analyses.

First, at this point in time, and given the variability of project development across the five cases, research contributions are mostly focused on interactions with networks and stimulating important conversations in the LTC sector. Although papers, tools and presentations were identified, it is too early to see policy or practice change as a result of these knowledge products. Interactions and conversations are highlighted here because of their importance in leading to action [28, 29]. In terms of interactions with networks, the projects were linked to end-users and the larger LTC community through direct partnerships and through the CLRI committees, both of which had access to their own networks of interested organisations and members. As noted by others, these interactions are important and necessary, and like ripples in a pond, lead to greater reach of the research findings [30]. In fact, a study of research contributions across 30 cases found that ﻿end-﻿user research partners facilitated the use of research findings through their interactions with external potential users [31]. Our analysis demonstrated that the ripple effect was indeed occurring. This effect can lead to direct incorporation of findings and to future practice changes being acceptable in the LTC community because of early awareness in the sector. Relatedly, novel conversations were taking place in the sector and at the Ministry level, exactly at a time when LTC sector reform is at the forefront, and thus these conversations lead to conceptual understandings of innovative solutions before action can take place. These conversations were also important for highlighting hidden provincial resources that, until now, had not received much attention.

The second significant finding is that the Steering and Management Committees represent an effective research-to-action platform to support the projects. Each of the Committees demonstrated that they had unique roles with respect to their relationship with the projects, and the analysis illustrated that these roles were being fulfilled. Further, these roles were seen as incredibly helpful by the research teams. This type of platform is different than what some scholars call ‘research knowledge infrastructure’, which can be characterised as technological or organisational resources, like electronic databases, data analysts or training programmes [32]. This research-to-action platform created by the CLRI actually represents a new model of knowledge brokering [33, 34] that has had little attention in the literature. The added-value of this platform is that it takes some of the burden of knowledge translation off the researcher, freeing up their time to concentrate on the science [35]. This was accomplished by making connections with the LTC community, by helping researchers frame their work for scalability, relevance and feasibility, by creating interdisciplinary teams, and by providing venues for dissemination – all of these activities contributed to better research and increased capacity (research capacity for trainees and knowledge translation capacity for researchers) for LTC systems-level research.

The third important finding is that the partnerships were beneficial and important. While some potential improvements were noted in the analysis, the qualities of a good relationship as identified by respondents (trust, good communication, mutually beneficial, challenging assumptions and leveraging opportunities) were shown to be generally characteristic of the project partnerships. These early and on-going relationships are important to enhance the relevance of the research, leading the way to utilisation of the findings [21, 36]. Not only are these partnerships important for the projects, but these linkages can support effective programmes of research. In this way, lack of grant funding success or lack of anticipated results do not jeopardise the strong relationships that have been forged. Indications are that such relationships have been formed in some of the cases. Systems-level partnerships for government-commissioned research, like the CLRI projects or the Collaborations for Leadership in Applied Health Research and Care in the United Kingdom, may benefit from recommendations based on our findings of early specification of partner/researcher expectations, continuous documentation of interactions, timeliness of conversations and responsiveness to user input. Gagliardi et al. [20] identified further enablers, including training/mentoring, forums for interaction and leadership.

A deeper, somewhat nuanced understanding of linking with end-users has recently emerged in the literature. The current assumption is that end-users ought to be partners throughout a research project. However, Kok et al. [31] found that research use occurred when users initiated research studies. Further, a critical finding was that use was more likely when a user who initiated a study and could potentially use the study findings remained involved in the research process: they supported potential use by others by activating their own external contacts, a process we saw emerging from our own data. In other cases, Kok et al. [31] found that only being involved in the interpretation of findings was critical for the use of research by users. These findings suggest that future studies might concentrate on specifying the stages of research during which potential users are engaged.

On a related note, the emerging tensions associated with user engagement bring to attention the need to unpack inclusiveness in the face of productivity in researcher–user relationships. One current line of thought is that user–researcher partnerships are effective because both parties bring a unique skillset to the table that, together, result in synergies influencing the research process, results and implementation efforts [37]. This view aligns with Pisani and Kok [38] in that different perspectives outside of academia contribute to research that is ‘socially robust’, or namely, useful. The authors write that engagement may “*…slow the process, but it will improve the result*” ([38] p. 7). An effective partnership may further require alignment at two other levels to support implementation. During the research process, efforts are needed to ensure that the user’s organisation, at the management level, is aligned with the project focus for subsequent “*institutional embedded*[ness]” [39]. Further, alignment ought to occur at the external level with other organisations and priorities [39], as was demonstrated in our study. Our findings, and those of others, point to a need for reflection on how to best organise and manage engagement.

This study is among the first to identify early, broadly-defined research contributions and related processes. These are important to identify because, “*even before formal knowledge outputs are designed, there may be uptake of emerging knowledge in practice…*” ([31] p. 5), and such formative assessments can assist in mid-course corrections and ‘alignments’. Nevertheless, the findings presented here need to be considered in light of certain study limitations. The most important challenge is identifying research contributions so early in the research process. As time goes on, it is likely that stronger project influences will be seen across the LTC community. In the interim, the use of multiple cases and sources of data contributed to the trustworthiness of the findings. Another challenge was ensuring that data come from a variety of individuals associated with the project. Traditionally, the strength of the focus group method is that recall and consensus are facilitated; the small numbers of individuals in the focus groups might not have allowed this strength to emerge. Generally, however, the continuity of participants at the two points of data collection supported a trusting relationship with the moderator to engage in the on-going conversation. There were some unheard perspectives, which might have contributed different views not expressed in this paper. Nevertheless, findings are based on multiple cases that demonstrate different characteristics that may support or hinder associated research outputs and contributions. As such, the findings may point to certain universal insights about the contributions of research that may be important for other settings or situations.

## Conclusion

This study has described some of the early contributions of five health systems-level LTC projects that go beyond the form of research products. These contributions range from the individual level, e.g. increased individual capacity, to the systems level via increased understanding of the broader system of LTC. The importance of end-user engagement throughout the research project was key among all research projects. This engagement was described as important in producing research that could inform policy change and health planning in LTC.

## Abbreviations

CLRI: Centre for Learning, Research and Innovation
LTC: long-term care
MOHLTC: The Ontario Ministry of Health and Long-term Care
